# Complete Genome Sequence of Salmonella enterica Serovar Enteritidis Myophage Mooltan

**DOI:** 10.1128/MRA.00187-19

**Published:** 2019-04-25

**Authors:** Jacob Chamblee, Chi Zeng, Chandler J. O'Leary, Jason J. Gill, Mei Liu

**Affiliations:** a Center for Phage Technology, Texas A&M University, College Station, Texas, USA; Queens College

## Abstract

Salmonella enterica serovar Enteritidis is a Gram-negative bacterium and one of the most common foodborne pathogens. Biocontrol using bacteriophage in food products or animals is one possible means by which pathogenic salmonellosis infection could be inhibited. Here, we report the complete genome sequence of the T4-like Salmonella Enteritidis myophage Mooltan.

## Abstract (precis)

Salmonella enterica serovar Enteritidis is a Gram-negative bacterium and one of the most common foodborne pathogens. Biocontrol using bacteriophage in food products or animals is one possible means by which pathogenic salmonellosis infection could be inhibited.

## ANNOUNCEMENT

Foodborne Salmonella enterica serovar Enteritidis infection is a regular and perpetual problem in the United States (1–3). As rising antibiotic resistance continues to represent a growing health care problem throughout the world, it is necessary to explore alternative means, including phage therapy, to control this bacterium (3, 4).

Phage Mooltan was isolated from mixed municipal wastewater collected in Brazos County, TX, in 2015 by using *S.* Enteritidis as a host. Host bacteria were cultured on tryptic soy broth or agar (Difco) at 37°C with aeration. Phage were cultured and propagated by the soft agar overlay method (5). Phage genomic DNA was prepared using a modified Promega Wizard DNA cleanup kit protocol, as described previously (6). Pooled indexed DNA libraries were prepared using the Illumina TruSeq Nano LT kit, and sequences were obtained from the Illumina MiSeq platform using the MiSeq v2 500-cycle reagent kit, following the manufacturer’s instructions, producing 697,877 reads for the index containing the phage genome. FastQC (http://www.bioinformatics.babraham.ac.uk/projects/fastqc/) was used for quality control of the reads. The reads were trimmed with FastX-Toolkit 0.11.6 (http://hannonlab.cshl.edu/fastx_toolkit/) before being assembled into a single contig at 42.6-fold coverage using SPAdes 3.5.0 (7). Contig completion was confirmed by PCR using primers (5′-GTTCCGTGAACAAGTGCTGA-3′ and 5′-ATTAGGTTGTGCTGGCGATT-3′) facing off the ends of the assembled contig and Sanger sequencing of the resulting product, with the contig sequence manually corrected to match the resulting Sanger sequencing read. GLIMMER 3.0 (8) and MetaGeneAnnotator 1.0 (9) were used to predict protein-coding genes, with manual correction for appropriate gene starts, and tRNA genes were predicted with ARAGORN 2.36 (10). Rho-independent termination sites were identified via TransTerm (http://transterm.cbcb.umd.edu/). Sequence similarity searches by BLASTp 2.2.28 (11) and conserved domain searches with InterProScan 5.15-54.0 (12) were used to predict protein function. All analyses were conducted using default settings via the CPT Galaxy (13) and WebApollo (14) interfaces (cpt.tamu.edu).

Mooltan has a 156,937-bp genome with a coding density of 92% and a GC content of 44.9%. Essential genes related to replication and recombination, as well as phage morphogenesis, were identified. Two tail spikes were identified, one of which is P22 gp19 like, suggesting use of the lipopolysaccharide O antigen as a binding receptor (15). An endolysin was identified that is predicted to contain an N-terminal peptidoglycan binding domain and is soluble due to the absence of an N-terminal signal anchor release (SAR) domain (16). Holin and spanin complexes, however, could not be reliably identified. Two selfish genetic elements were identified in the genome, an intein in the large terminase subunit and a GIY-YIG homing endonuclease. The terminase is predicted to utilize headful packaging by homology of the terminase large subunit (TerL) with other characterized headful terminases. The tail tape measure protein was not identified.

Many characteristic T4 genes were identified in the Mooltan genome, and this genome was found to be syntenic (have the same gene order) with phage T4, with *rIIA* as the first gene of the genome. Additionally, Mooltan carries a P22 gp17-like superinfection exclusion protein. In P22, gp17 is necessary to counteract the Fels-2 prophage superinfection exclusion system (17).

### Data availability.

The genome sequence of phage Mooltan was submitted to GenBank under accession number MH688040. The associated BioProject, SRA, and BioSample accession numbers are PRJNA222858, SRR8556780, and SAMN10904482, respectively.
